# *In Vitro* Cytotoxicity of Folate-Silica-Gold Nanorods on Mouse Acute Lymphoblastic Leukemia and Spermatogonial Cells

**DOI:** 10.22074/cellj.2019.5691

**Published:** 2018-11-18

**Authors:** Neda Eslahi, Ali Shakeri-Zadeh, Khadijeh Ashtari, Vahid Pirhajati-Mahabadi, Tahereh Tohidi Moghadam, Ronak Shabani, Kamran Kamrava, Zahra Madjd, Chad Maki, Hamid Reza Asgari, Morteza Koruji

**Affiliations:** 1 Department of Anatomical Sciences, School of Medicine, Iran University of Medical Sciences, Tehran, Iran; 2 Department of Medical Physics, School of Medicine, Iran University of Medical Sciences, Tehran, Iran; 3 Cellular and Molecular Research Center, Iran University of Medical Sciences, Tehran, Iran; 4 Department of Medical Nanotechnology, Faculty of Advanced Technologies in Medicine, Iran University of Medical Sciences, Tehran, Iran; 5 Neuroscience Research Center, Iran University of Medical Sciences, Tehran, Iran; 6 Department of Nanobiotechnology, Faculty of Biological Sciences, Tarbiat Modares University, Tehran, Iran; 7 Clinical Nanomedicine Laboratory, ENT-Head and Neck Research Center, Hazrat Rasoul Akram Hospital, Iran University of Medical Sciences, Tehran, Iran; 8 Oncopathology Research Center and Dep Pathology, Faculty of Medicine Iran University of Medical Sciences, Tehran, Iran; 9 VetCell Therapeutics, Daimler St, Santa Ana CA, USA

**Keywords:** Acute Lymphoblastic Leukemia Cells, Cytotoxicity, Folic Acid, Gold Nanorods, Spermatogonial Cells

## Abstract

**Objective:**

The purpose of this study was to evaluate *in vitro* cytotoxicity of gold nanorods (GNRs) on the viability of spermatogonial cells (SSCs) and mouse acute lymphoblastic leukemia cells (EL4s).

**Materials and Methods:**

In this experimental study, SSCs were isolated from the neonate mice, following enzymatic digestion and differential plating. GNRs were synthesized, then modified by silica and finally conjugated with folic acid to form F-Si-GNRs. Different doses of F-Si-GNRs (25, 50, 75, 100, 125 and 140 µM) were used on SSCs and EL4s. MTT (3-(4,5-dimethylthiazol-2-yl)-2,5-diphenyltetrazolium bromide) proliferation assay was performed to examine the GNRs toxicity. Flow cytometry was used to confirm the identity of the EL4s and SSCs. Also, the identity and functionality of SSCs were determined by the expression of specific spermatogonial genes and transplantation into recipient testes. Apoptosis was determined by flow cytometry using an annexin V/propidium iodide (PI) kit.

**Results:**

Flow cytometry showed that SSCs and EL4s were positive for Plzf and H-2kb, respectively. The viability percentage of SSCs and EL4s that were treated with 25, 50, 75, 100, 125 and 140 µM of F-Si-GNRs was 65.33 ± 3.51%, 60 ± 3.6%, 51.33 ± 3.51%, 49 ± 3%, 30.66 ± 2.08% and 16.33 ± 2.51% for SSCs and 57.66 ± 0.57%, 54.66 ± 1.5%, 39.66 ± 1.52%, 12.33 ± 2.51%, 10 ± 1% and 5.66 ± 1.15% for EL4s respectively. The results of the MTT assay indicated that 100 µM is the optimal dose to reach the highest and lowest level of cell death in EL4s and in SSCs, respectively.

**Conclusion:**

Cell death increased with increasing concentrations of F-Si-GNRs. Following utilization of F-Si-GNRs, there was a significant difference in the extent of apoptosis between cancer cells and SSCs.

## Introduction

Cancer is a disease that grows fast and out of control which is capable of spreading and growing anywhere in the body. The incidence of childhood cancer is annually 141 per million in the USA. In Iran, Cancer is the third cause of death (1). Childhood cancer is a treatable disease due to the development of chemo- and radiation therapies, but long-term survivors may be suffering from infertility. 

Cytotoxic factors and radiation impair spermatogenesis cause oligospermia or azoospermia as well as genetic damage in sperm. An approach to overcome this problem in a child with leukemia or other metastatic cancers is the use of fresh or cryopreserved testicular cells that are not infected with cancer cells (2). After treating cancer in these patients, spermatogonial stem cell (SCC) transplantation into the testes can potentially restart spermatogenesis (3). 

The number of transplanted stem cells is critical for the effectiveness of the transplantation technique (4) and stem cell enrichment for transplantation may be necessary (5, 6). On the other hand, with leukemia or any kind of childhood metastatic cancer, there is a risk of contamination of SSCs with cancer cells. In addition to SSC manipulation (enrichment, purification and cryopreservation), decontamination of cancer cells from testicular suspension may be necessary and unavoidable for patients at risk before autotransplantation (7, 8).

Cell sorting is a good method to decontaminate cancer cells from normal cells. These approaches include immunomagnetic (MACS) and immunofluorescent (FACS)-based strategies, but sorting does not properly remove contaminated cells in all cases (9, 10). Shabani et al. (7, 11) applied cisplatin before cell sorting to eliminate contaminated malignant cells from germ cells. They discovered that treatment with effective doses of cisplatin was useful in the isolation of SSCs from tumour cells. As a suggestion, applying gold nanoparticles (NPs) may be beneficial to remove malignant cells before cell sorting. 

Gold NPs play a great role in cancer treatment because their exposure to UV and infrared radiation destroys cancer cells through the production of heat. They also increase the lifetime and delivery of drugs such as anticancer drugs that are very insoluble or unstable in the biological environment (12). Therefore, gold NPs may be used in chemotherapy, photothermal therapy (PTT), radiation therapy (RT) and photodynamic therapy (PDT) (13, 14). 

Examples of GNPs are gold nano cages (GNCs), gold nanorods (GNRs), and gold nanospheres (GNSs). Among them, GNRs have been shown to be the most efficient NPs at absorbing near-infrared (NIR) light and converting that energy to heat (15) which could be at least 6X more effective than gold nanospheres or nanoshells (16). Nowadays, for selectively targeting cancer cells, a specific binding site on the surface of the cell, such as a receptor, is used (17). A more effective and active targeting system is needed to increase intracellular uptake of NPs containing drugs by cancer cells in the tumor site. Different ligands such as vitamins, hormones and monoclonal antibodies against tumor cell-specific receptors have been loaded on the surface of NPs to deliver them into cells via receptor- mediated endocytosis (18). Among them, the vitamin folic acid (folate) has been extensively used as the best target for different anti-cancer drugs (17, 19). In order to enhance stability of gold NPs thermodynamically and chemically, silica coating has been used (20, 21).

Xia et al. (22) used F-Si-GNRs on A549 cells and HeLa cells. They show that uptake of NPs into HeLa cells via receptor-mediated endocytosis was more efficient than folate receptor-deficient A549 cells. Huang et al. (23) used F-Si-GNRs on MGC803 gastric cancer cells. Also, Gao et al. (24) showed high uptaking occurred for F-Si- GNRs by HepG2. 

In this study, we performed the MTT (3-(4,5-dimethylthiazol-2-yl)-2,5-diphenyltetrazolium bromide) proliferation assay to evaluate the cytotoxicity of F-Si-GNRs on SSC and EL4 cells. To achieve an effective dose and incubation time with F-Si-GNRs, we examined different doses of F-Si-GNRs at different times on cancer cells and germ cells.

## Materials and Methods

### Materials for synthesis and surface modification of gold nanorods

In this experimental study, HAuCl_4_·3H_2_O, NaBH_4_, Ascorbic acid, Hexadecyl trimethyl ammonium bromide (CTAB), AgNO_3_, Tetraethylorthosilicate (TEOS) and Folate were purchased from Sigma (Germany). Phosphate buffered saline tablet (PBS) and also Sodium acetate was obtained from Merck (USA). Glassware was thoroughly cleansed with a dilute sulfochromic acid solution and detergent,followed by rinsing with de-ionized (DI) water. 

### Preparation of Au seeds and nanorods

GNRs were synthesized via sequential seed-mediated growth method, as described elsewhere (23). In summary, small spherical gold NPs (seeds) were prepared by mixing aqueous solutions of HAuCl_4_·3H_2_O (250 µL, 0.01 M) and CTAB (7. 5 mL, 0.095 M), followed by immediate addition of an ice-cold NaBH4 solution (600 µL, 0.01M). The reactants were mixed by rapid inversion for two minutes and kept undisturbed at room temperature for a minimum of 2 hours. Then the growth solution was accumulated by sequential addition of CTAB (9. 5 mL, 0.095 M), HAuCl_4_·3H_2_O (400 µL, 0.01 M), AgNO_3_ (60 µL, 0.01 M) and ascorbic acid (64 µL 0.10 M) solutions, followed by mixing with seed particles (40 µL). It takes several hours for termination of the reaction and formation of rod-shaped nanostructures. 

### Purification of gold nanorods 

The unreacted gold ions and excess cationic surfactant (CTAB) were removed by centrifugation (14,000 rpm, 7 minutes). The sediment was diluted with distilled water, then the purified sample sonicated for several minutes to redisperse the nanorods. Prior to surface modification with silica, absorbance intensity of the stock GNRs was adjusted to optical density (OD). 

### Surface modification of gold nanorods

Ten milliliters of purified GNRs were redispersed in ethanol, and the pH was adjusted to 10 using ammonia. The suspension was sonicated in a water bath for several minutes. 20 µl of TEOS was diluted to 1 mL with ethanol which was sequentially added to GNRs (20 µL each time) at 30 min intervals. The solution was vigorously stirred overnight. Silica-coated GNRs (Si-GNRs) were purified by centrifugation at 3,500 rpm for 30 minutes followed by several rounds of washing with water and ethanol. 1.5 mg folate was dissolved in 2 mL dimethyl sulfoxide (DMSO). For each 10 mL suspension of GNRs in ethanol, 250 µL of folate solution was used. Samples were further purified and used for characterizations.

### Equipment for characterization 

Characteristic surface plasmon resonances of GNRs were recorded in the wavelength region of 400 to 900 nm, using a Perkin Elmer spectrophotometer (Lambda 25). For Fourier- transform infrared spectroscopy (FTIR) analysis, samples of bare GNRs, silica, and folic acid modified GNRs were made into a dry powder by a lyophilizer (LYSFME-Snijders scientific). Spectra were recorded on a NICOLET IR 100 (FT-IR) and reported in the range of 500-3,800 cm^-1^.

For transmission electron microscopy (TEM) characterization, purified and surface modified GNRs were deposited on carbon-coated copper grids and imaged utilizing TEM (LEO 906, Zeiss). 

The dynamic light scattering (DLS) was performed by Brookhaven 90Plus Nanoparticle Size Analyzer to identify the effective diameter and size distribution of GNRs. The surface charge of F-Si-GNR was measured with Zeta potential measurements in water (NICOMP 380ZLS Zeta potential/Particle sizer).

### Animals

In this study, 120 neonatal mice between 3-6 days old were used. These animals were obtained from the Experimental and Comparative Studies Center of Iran University of Medical Sciences (IUMS). The animals were housed in cages at 22-25°C with a 12 hours: 12 hours cycle and given free access to food and water at all times. All studies were performed in accordance with the Ethical guidelines set by the “animal care and use committee (ACUC), Iran University of Medical Sciences” (code: IR.IUMS.rec.1394-01-1172-5884). 

### Isolation and culture of spermatogonial stem cells 

Testes were collected aseptically from 3-6-day-old mice. First of all, testes were decapsulated, then minced and suspended in Dulbecco’s Modified Eagle Medium (DMEM, Life Technologies, Carlsbad, CA, USA) supplemented with 1. 37 g/L NaHCO3 (Sigma-Aldrich, St Louis, MO, USA), penicillin (100 IU/mL), streptomycin (100 µg/m), gentamycin (40 µg/mL) and single-strength nonessential amino acids, (all from Life Technologies).

Testicular cells were isolated according to our previous study (25). In summary, testes fragments were digested in DMEM containing 0.5 mg/mL collagenase/dispase, 0.5 mg/mL Trypsin, and 0.05 mg/mL DNAse (all from Sigma-Aldrich), for 30 minutes at 37°C. The interstitial cells were removed by washing in DMEM medium. The second step of digestion was performed by adding the same fresh enzyme solution in DMEM media as described above. After cell separation and filtration through 70-µm nylon filters, cell viability was determined and the harvested cells were used for cell culture. Myoid and Sertoli cells were also separated by overnight differential plating in DMEM containing 5% fetal calf serum (FCS). Then the harvested spermatogonia were cultured in DMEM containing 5% FCS and 10 ng/mL GDNF for 2 weeks. The cells were incubated at 32°C, 5% CO_2_, approximately 85% humidity, and the medium was refreshed every 2-3 days. 

### Reverse transcription polymerase chain reaction

This study was performed in following groups: cells obtained from enzyme digestion, cells derived from cultured colonies after two weeks, and mouse testis tissue as a positive control. The expression of spermatogonial genes was determined based on previous animal studies. RNA was extracted using a standard RNA extraction kit (Qiagen, Germany) per the manufacturer’s instructions. The RNA was examined for purity and integrity by a 260/280 nm ratio measurement. In the reverse transcription reaction, 1 µg of total RNA was used with QuantiTect® Reverse Transcription Kit (Qiagen) per the manufacturer’s instructions. 

The primers specific for GDNF family co-receptor α1 (*Gfrα-1*), promyelocytic leukemia zinc-finger (*Plzf*), *Itgß1*(ß1-integrin) , *Itgα6 (α6-integrin), VASA* homologue (*Mvh*), octamer-binding transcription factor 4 (*Oct4*) and *Gapdh* genes were designed using mouse sequences (Gene Bank) and Gene Runner software (version 3. 02, Hastings Software Inc, USA) as shown in Table 1. *Gapdh* was a housekeeping gene. Reverse-transcription polymerase chain reaction (RT-PCR) was performed using the primers, the prepared complementary deoxyribonucleic acid (cDNA) and PCR Master Mix 2X kit (Fermentas, Germany) , under the following conditions: 95°C for 3 minutes, followed by 35 cycles at 95°C for 30 seconds, under specific annealing temperature for each primer (*Plzf, 55°C; Oct4, 60°C; Gfrα-1. 52°C; Vasa, 62°C; Itgα6, 52°C; Itgß1, 55°C and Gapdh, 60°C*) for 45 seconds, 72°C for 60 seconds, and a final extension of 72°C for 10 minutes. 

PCR products were separated by resolving 1 µL of each sample on a 1.2% agarose gel, and electrophoresis was performed with Tris-Borate-EDTA (TBE) 1x loading buffer (Sigma-Aldrich, Germany) at a voltage of 95 for 45 minutes. The gels were stained with 0.1 µg/mL Gel Red™ (Biotium Inc, USA) and we used Gel Logic for visualization of bands (Carestream Health Inc., Rochester, NY, USA). 

### Confirmation of the spermatogonial stem cells 

For functional confirmation, spermatogonial stem cells were labeled with DiI (Invitrogen, Carlsbad, CA, USA) and DAPI (Sigma, Germany), then injected into the seminiferous tubules of busulfan-treated mice. 5 mg/ ml DMSO was used as a solvent for preparation of the busulfan dosage. Also an equal volume of warm (40°C) distilled water was added to above solution to prevent precipitation of DMSO. A single dose of busulfan (40 mg/ kg) was injected intraperitoneally in the NMRI mice (25). 

Mice weighing 25 g were treated with 400 µl of the final busulfan solution. 4 weeks after treatment with busulfan, mice were devoid of most endogenous germ cells. The mice (n=5) were anesthetized with intraperitoneal (i.p.) injection of ketamine hydrochloride 10% (Rotexmedica, Germany) (100 mg/kg) and xylazine 2% (Alfasan, Holland, 10 mg/kg). Then spermatogonial cells (SSCs, 106/ml) were resuspended in 10 µl DMEM/F12 and injected directly through the efferent ductus and into the seminiferous tubules of the busulfan-treated mice. Seminiferous tubules were visualized by addition of trypan blue in the injection media. 8 weeks after transplantation, survival and proliferation rates of cells were estimated by fluorescent microscopy (type CH_2_, 4009 magnifications; Olympus, Japan). 

**Table 1:** The sequence of the designed primers used for reverse transcriptase polymerase chain reaction


Genes	Primer sequences (5´-3´)	Annealing temperature (˚C)	Size (bp)

*Igα_6_*	F: CTC AGA ATA TCA AGC TCC CT	60	148
	R: AAA CAC TAA TAG AGC CAG CA		
*Gfrα_1_*	F: AAT TGT CTG CGT ATC TAC TGG	60	130
	R: ACA TCT GAT ATG AAC GGG AC		
*Igβ_1_*	F: GAC ATT ACT CAG ATC CAA CCA	60	115
	R: AGG TAG TAG AGA TCA ATA GGG T		
*Oct_4_*	F: GAA CTA GCA TTG AGA ACC GT	60	115
	R: CAT ACT CGA ACC ACA TCC TTC		
*Plzf*	F: CCC GTT GGG GGT CAG CTA GAA	61	137
	R: CTG CAA GGT GGG GCG GTG TAG		
*Mvh(Vasa)*	F: GAT AAT CAT TTA GCA CAG CCT C	59-61	149
	R: GTC AAC AGA TGC AAA CAC AG		
*Gapdh*	F: CAA CTC CCA CTC TTC CAC TT	60	125
	R: GCA GCG AAC TTT ATT GAT GGT A		


### Culture and tumourigenicity confirmation of El-4 cell line 

We commercially obtained the mouse acute lymphoblastic leukemia cell line EL4 from Pasteur Institute (Tehran, Iran). The EL4 cells were cultured in HEPES DMEM/F12 (Gibco, USA), 2% fetal bovine serum (Gibco, USA), 1% penicillin (Invitrogen, UK), and 1% streptomycin (Invitrogen, UK). For Confirmation of tumorigenicity and induction of the xenograft tumor model, 5×10^4^ EL4s in 10 µl medium were transplanted through the efferent ductus and into the seminiferous tubules of azoospermia busulfantreated male NMRI mice (20-30 g) (26). The shape and thickness of each tumour was evaluated eight weeks after EL4 cell injection. Both testes were surgically removed and processed for histological examination. 5 µm thickness sections were stained with hematoxylin and eosin (H&E). The volume of tumors (V_t_) was estimated in the formula: V_t_=p (b2×a)/6 (b and a are the minimum and maximum diameters in millimeters respectively). 

### Flow cytometry

We used flow cytometry to confirm the identity of the EL4s and SSCs. Isolated SScs (10^6^ per 100 µl PBS) were incubated in the dark for 30 minutes at 4°C with PLZF monoclonal antibody (ebiosciences, 53-9320-82, 1: 50), Then, the cells were washed with PBS (three times). Also, the EL4 cells (10^6^ per 100 µl PBS) were incubated with a FITC-conjugated mouse anti-*H-2kb* monoclonal antibody (ebiosciences; 553569, 1: 50). 

### Experimental groups and MTT assay 

In this study, EL4s and SSCs were divided into five groups: control (medium without F-Si-GNRs) and experimental groups, with cells distributed in a 96-well plate at a cell density of 15×10^3^ cells per well in the different concentrations of F-Si-GNRs (25, 50, 75, 100, 125, and 140 µM) for different incubation periods (6, 12 hours). We performed the MTT (3-(4,5-dimethylthiazol2- yl)-2,5-diphenyltetrazolium bromide proliferation assay to determine the toxicity of F-Si-GNRs. After centrifuging the cells, washing was done with PBS. Then 100µl of MTT solution [MTT tetrazolium salt (5 mg/ ml)] was added to each well and incubated for 3-4 hours, followed by centrifugation of the solution and removal of the supernatant. Next, 100 µl of DMSO was added to the wells, and plates were shaken for 10 minutes in a microplate shaker before observation with the ELISA reader at 570 nm. 

### Transmission electron microscopy

For TEM technique, SSCs and FL4 cells were washed with PBS, then 2.5% glutaraldehyde was used as a primary fixation for 2 hours. For removal of free glutaraldehyde, the cells were rinsed 2-3 times with PBS. Then, 1% osmium tetroxide was used as a secondary fixation for 1.5 hours. The cells were dehydrated in acetone (50, 70, 90, 100%), infiltrated by resin and finally embedded in pure resin (Epon 812, TAAB, UK). Semi-thin (500 nm) and thin (50 nm) sections were performed for light and electron microscopy respectively. Thin sections were transferred on the 200-mesh uncoated grids and stained with uranyl acetate and lead citrate before imaging with TEM (LEO 906; Zeiss). It should be noted that for GNR imaging, NPs were deposited on carbon-coated copper grids directly. 

### Apoptosis evaluation in SSCs and EL4 cells after treatment with F-Si-GNRs

In this study, we used an optimal mean dose of F-Si- GNRs (100 µM) for 6 hours. The apoptosis was measured using annexin V-fluorescein isothiocyanate (FITC) apoptosis detection. At first, the cells were plated at a density of 200,000 cells/well in 24-well plates. The cells were washed with PBS and then resuspended in annexin binding buffer. Then cells were incubated with annexin-FITC/PI in the dark for 15 minutes. In the next step, reasonable results were obtained by flow cytometric counting of viable cells. Viable cells were negative for both PI and annexin V-FITC; necrotic cells were positive for PI and negative for annexin-V-FITC. early apoptotic cells were positive for annexin-V-FITC and negative for PI, whereas late apoptotic cells were positive for both annexin-V-FITC and PI. 

### Statistical analysis

Data have been presented as the mean ± SD with at least three biological independent repeats. Differences between groups were assessed by One-way ANOVA using the SPSS version 25 software (SPSS Inc., Chicago, IL, USA). The difference between groups was considered statistically reliable if P≤0.05. 

## Results

### Expansion and characterization of spermatogonial cells 

**Fig.1:**
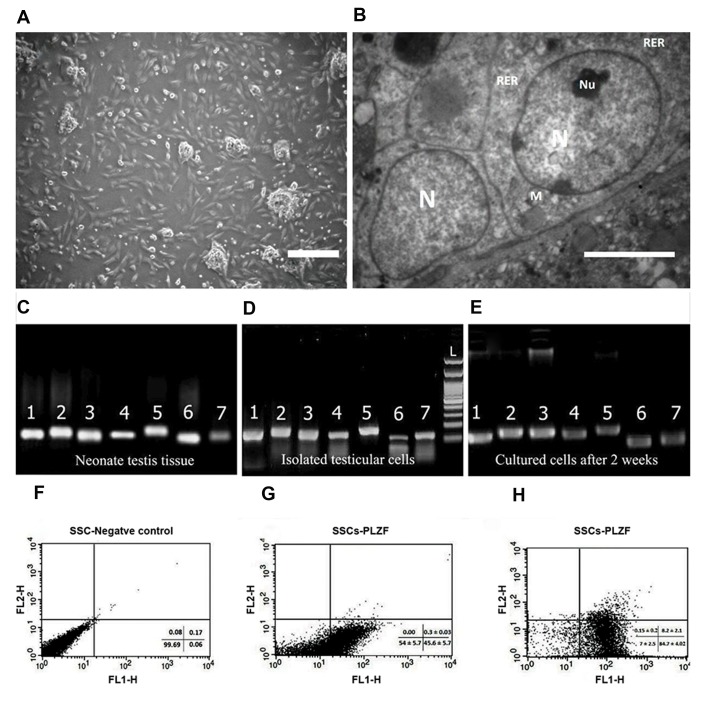
Spermatogonial cells characterization. **A**. The morphology of a spermatogonial-derived cluster formed from the culturing of spermatogonial cells after 24 hours (scale bar: 200 µm), **B**. Representative transmission electron micrographs from spermatogonial cells (SSCs) clusters (scale bar: 5 µm). The heterochromatin nucleus (N), eccentric small compact and highly reticulated nucleoli (Nu), Rough endoplasmic reticulum (RER) and very high mitochondria (M) were observed in cells. Reverse transcription polymerase chain reaction (RT-PCR) was used to determine the expression of specific spermatogonia and germ cell markers in **C**. Neonate testis tissue (fresh tissue without enzymatic digestion), **D**. Cultured cells after the first day and **E**. Two weeks of culture. 1; Oct4 (129 bp), 2; Itgα6 (148 bp), 3; Plzf (137 bp), 4; Gfrα1 (130 bp), 5; Mvh (Vasa, 149 bp), 6; Itgß1 (115 bp), 7; Gapdh (125 bp). Flow cytometric analysis of spermatogonial cells: **F**. Spermatogonial negative control, **G**. The PLZF positive spermatogonial cells at the end of the first week were 45.63 ± 5.71%, and **H**. At the end of the second week was 84.68 ± 4.02%.

Following the enzymatic digestion of the testicular tissue, the SSCs were isolated and cultured in DMEM/ F12 medium containing 5% FBS for 2 weeks. In order to increase the proliferation of the cells, GDNF (10 ng/ ml) was added to the culture medium. After 24 hours (Fig .1A), the SSCs formed colonies, and after 72 hours the platform was covered with cluster colonies. About 2-3 days after the primary culture, the cluster of germ cells appeared on a feeder layer. These were clumpy and had individually recognizable cells. They were then enzymatically dispersed and subcultured. During 2 weeks of culture, SSCs could start the formation of new clusters. The addition of GDNF in culture resulted in a significant improvement in SCC proliferation. In transmission electron microscop, the heterochromatin nucleus (N), eccentric small compact and highly reticulated nucleoli (Nu) and very high mitochondria (M) were observed in SSCs clusters (Fig .1B). 

In order to confirm the identity of spermatogonial stem cells, the expression of specific SCC markers was analyzed in the fresh tissue (without enzymatic digestion), isolated testicular cells (after first day of culture) and cultured cells (after 2 weeks of culture) by RT-PCR. As shown in Figure 1C-E, specific genes of SSCs are expressed in all samples (*Oct4, Itgα6, Plzf, Gfrα1, Mvh, Itgß1,* and *Gapdh* as a housekeeping gene). 

The results of flow cytometry show that the average amount of *Plzf* expression in SSCs at the end of the first and second weeks of culture were 45.63 ± 5. 71% and 84.68 ± 4. 02%, respectively (Fig .1F-H). 

### Culture of the EL-4 cells and characterization

**Fig.2:**
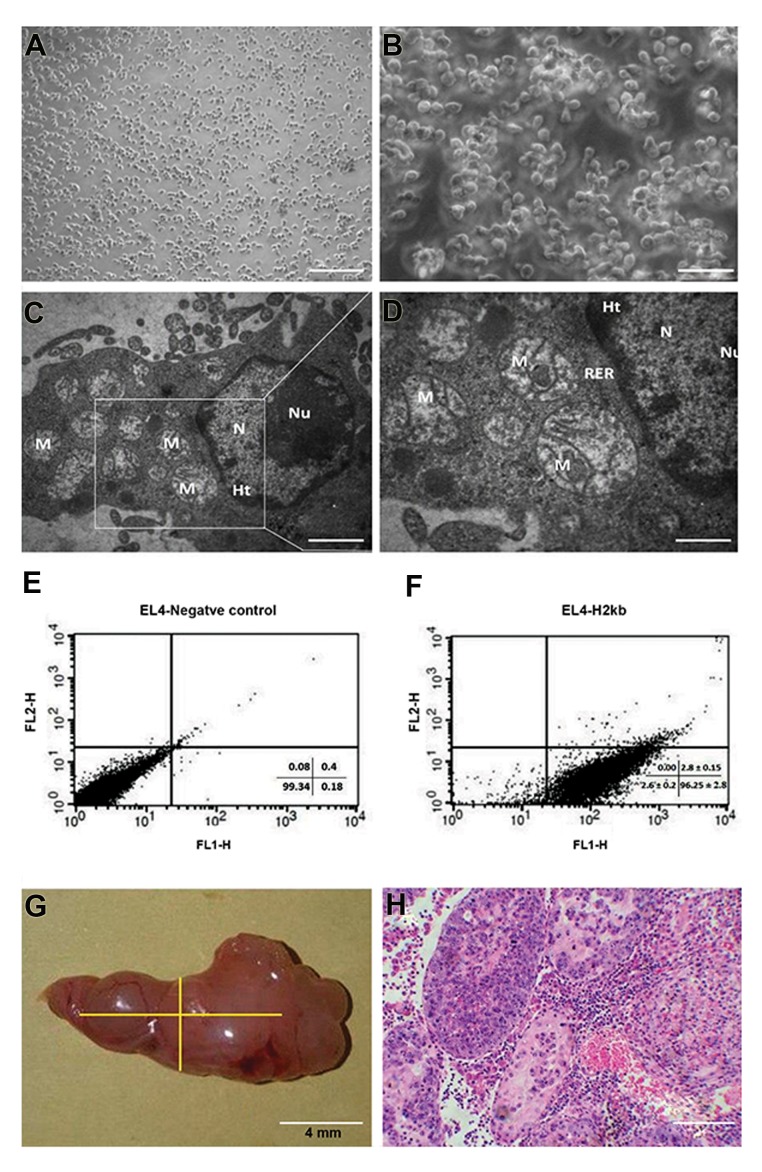
El-4 cells charectrization. A, B. Representative phase contrast images (scale bar, A: 200 µm, B: 50 µm), C, D. Transmission electron micrographsfrom EL4s (scale bar, C: 2 µm, D: 1 µm). These cells formed irregular margins. Spherical mitochondria were found in relatively high numbers. Inaddition, the nucleus of some cells had marginal heterochromatin. Nucleus (N), Nucleolus (Nu), Mitochondria (M), Rough endoplasmic reticulum(RER) and Heterochromatin (Ht), E, F. Flowcytometry analysis of EL-4 cells labeled for H-2Kb. The H-2Kb positive EL4s were 96.25 ± 2.81%, and G, H. tumor formation of El-4 cells in azoospermic recipient mouse model. In this model, 50,000 EL4s were transplanted, G. A testicular tumor formed 8 weeks after transplantation of EL4s (tumor size: 142 mm^3^) in recipient testis, H. Histological section of tumour formed from EL4 cells stained with H&E (scale bar: 50 µm).

Tumor cells were purchased from the Pasteur Institute (Tehran, Iran) after the fourth passage and cultured in DMEM/F12 medium containing 2% FBS. The cells were cultured in suspension and passaged every 48 hours. The margins of these cells were irregular. It should be noted that these cells don’t form colonies and have a high proliferation rate (Fig .2A, B). The ultrastructural characteristics of EL4s were examined via TEM. The nucleus and cytoplasm had an irregular shape. The cytoplasm was characterized by organelles, eg, mitochondria, rough endoplasmic reticulum. A large number of spherical mitochondria were found (Fig .2C, D). EL4s cells were confirmed by *H-2kb* monoclonal antibodies, respectively. The results of flow cytometry show that about 96.25 ± 2.81% of EL-4 cells expressed *H-2kb* (Fig .2E, F). 

### Tumourigenicity confirmation of EL-4 cells 

In order to confirm tumorigenicity, 5×10^4^ EL4 cells were transplanted through the efferent ductus and into the seminiferous tubules of azoospermia mice. After 8 weeks, the shape and thickness of any tumours were evaluated. Histological evaluations showed that after 8 weeks, a tumor had formed in 70% of the mice. The volume of tumours (V_t_) was estimated in the formula: V_t_=π (b2×a)/6 where b and a are the minimum and maximum diameters in millimeters, respectively. The average tumor size was 142 mm^3^ (Fig .2G). After 8 weeks, we observed that leukemic cells had infiltrated the interstitial tissue. These cells were polygonal with spherical nuclei (Fig .2H). The results showed that tumorigenicity of EL4 cells was restricted to testicular tissue. 

### Synthesis and characterization of F-Si-GNR

Surface plasmon resonance bands of GNR were monitored in the visible and NIR region, representing oscillation of the conduction band electrons along the short and long axis of GNRs. The appearance of a strong longitudinal surface plasmon resonance (LSPR) band around 798 nm, along with a transverse SPR band of weaker intensity around 512 nm is characteristic of formation of nanostructures with rod morphology. Changes in the SPR bands were also monitored upon formation of a silica layer around nanostructures. Stability of GNRs was checked in ammonia and ethanol, prior to interaction with tetraethyl orthosilicate (data not shown). Upon addition of TEOS, the longitudinal surface plasmon absorption band experienced a decrease in intensity; whereas the transverse surface plasmon absorption band did not undergo any remarkable changes. Due to the sensitivity of SPR bands to trace changes in the local environment, alterations in the intensity or wavelength position of the bands could be attributed to the interaction of the nanostructures with molecules. Hence, a decrease of longitudinal LSPR band intensity of GNRs upon interaction with TEOS represents the formation of a silica layer around the nanostructures. Such a type of coating is considered to be a useful strategy in replacement/coating of the cationic surfactant (i. e. CTAB), enabling application of GNRs as biocompatible platforms in a variety of biomedical approaches. Coating of GNRs with a very thin silica film (2.56 ± 0.62 nm in this study) improves the colloidal stability of the nanorods by reducing aggregation and allows for shape stability as well as surface modification. Furthermore, silica is porous and can be feasibly loaded with molecules of interest such as chemicals, drugs, dyes, or imaging agents either via physical adsorption or covalent attachment. 

Comparison of both of the characteristic SPR bands of GNRs after 30 minutes and 11 hours showed that within a typical range of concentration of TEOS, there is no change in the thickness of the silica layer over the nanostructures. Furthermore, the interaction of silica coated GNRs with folate shows a decreased intensity of both transverse and LSPR absorption bands, representing physisorption of folate onto the matrix of silica coated GNRs. 

**Fig.3:**
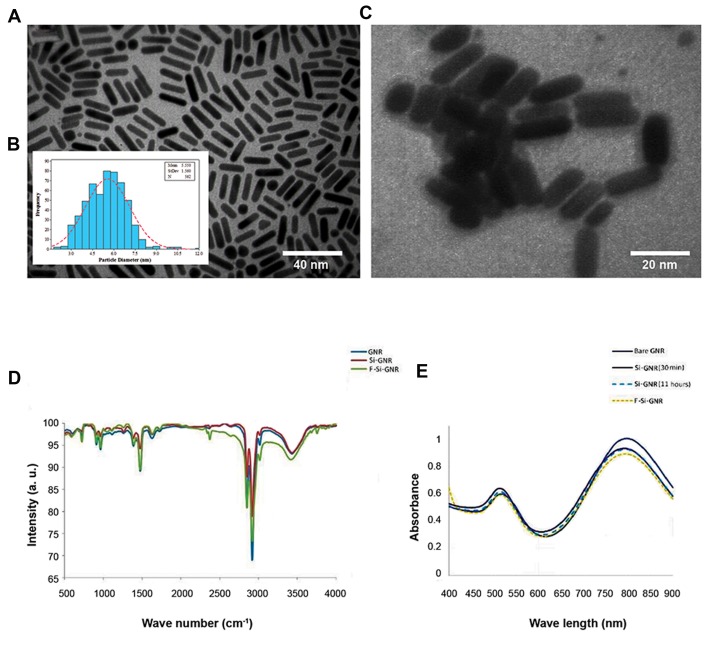
Analysis of the gold nanoparticles (GNRs). A. Transmission electron microscopy images of purified GNRs, B. Inset size distribution histogram (an average of 562 nanoparticles), C. Silica-coated GNRs. The thickness of silica layer was 2.56 ± 0.62 nm, D. Characteristic SPR bands of GNRs, before and after surface modification with silica and folate, and E. FTIR spectra of GNRs, silica coated GNRs (Si-GNR) and folic acid modified Si- GNR (F-Si-GNR).

We analysed TEM images of the GNRswith a size distribution histogram (an average of 562 NPs) (Fig .3A). An average diameter of 5.55 ± 1.56 nm was determined from the statistical analysis of the TEM images (Fig .3B). The images clearly show the formation of the rod morphology as well as a coating of the silica layer around GNRs (Fig .3C). Based on TEM images, the size of the nanostructures was 20.43 ± 2.18 nm in length and 5.55 ± 1.56 nm in width. The thickness of the silica layer coating around the nanostructures was 2.56 ± 0.62 nm.

Analysis of FTIR spectra for silica coated GNRs before and after physisorption of folic acid is shown in Figure 3D. A glance at the figure shows that upon modification of silica coated GNRs with folic acid, the spectral features have been changed. Folic acid is composed of p-aminobenzoic acid, glutamic acid, and a hetero-bicyclic pteridine that band between 1475 and 1500 cm^-1^. This is attributed to the characteristic absorption band of the phenyl and PT ring. Apart from the displacement in vibrations related to carbonyl group (1712 cm^-1^) and C=C (1388. 49 cm^-1^), the characteristic vibrational bands of folic acid for the phenyl and pterin ring (around 1478 cm^-1^), the OH carboxylic of glutamic acid moiety and the NH group of the pterin ring, (stretching in the range of 3500-3700 cm^-1^), depicts adsorption of folic acid molecules onto the matrix of silica-coated GNRs (Fig .3E). 

**Fig.4:**
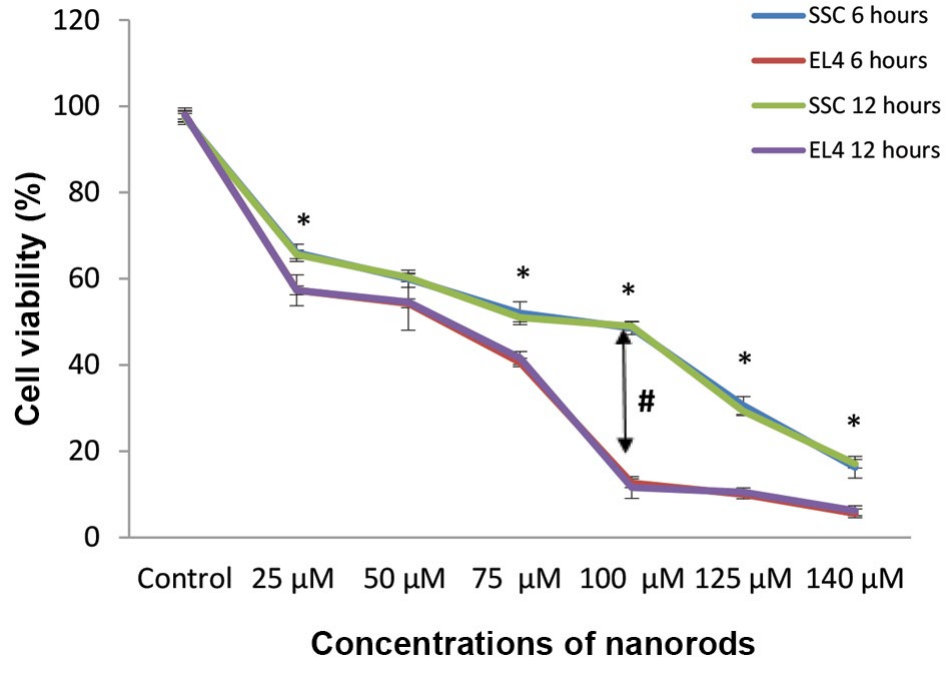
El-4 and spermatogonial stem cells viability at varying concentrations of nanorods (6, 12 hours). Cell death rates in EL4 cells were higher than SSCs, especially in 100 µM of GNRs, but there weren’t significant differences between 6 and 12 hours incubation periods. In each dosage category, there aren’t significant differences between groups 6 and 12 hours. The diagram shows that the optimal mean dose for highest cell death in EL4s and lowest in SSCs is 100 µM of GNRs. In each dosage category. *; There are significant differences between El4s and SSCs groups.

Optimal dosages and duration of F-Si-GNR for EL4s and spermatogonial stem cells was assessed. The survival of EL4s and SSCs after treatment with F-Si-GNR was assessed using the MTT test. The concentrations of F-Si- GNR tested ranged from 25, 50, 75, 100, 125 and 140 µM for different incubation periods (6, 12 hours). The percent viability of SSCs and EL4s that were treated with 25, 50, 75, 100, 125 and 140 µM of GNRs was 65.33 ± 3.51%, 60 ± 3.6%, 51.33 ± 3.51%, 49 ± 3%, 30.66 ± 2.08% and 16.33 ± 2.51% for SSCs and 57.66 ± 0.57%, 54.66 ± 1.5%, 39.66 ± 1.52%, 12.33 ± 2.51%, 10 ± 1% and 5.66 ± 1.15% for EL4s respectively. Given that there were not significant differences between 6 and 12 hour incubation periods, we chose 6 hours for incubation. It means that cell death increased with an increase in the quantity of GNRs. The results show that the optimal mean dose for the highest cell death in EL4s and lowest in SSCs is 100 µM of GNRs (Fig .4). 

### Ultrastructure and apoptosis evaluation in SSCs and EL4 cells after treatment with F-Si-GNR 

**Fig.5:**
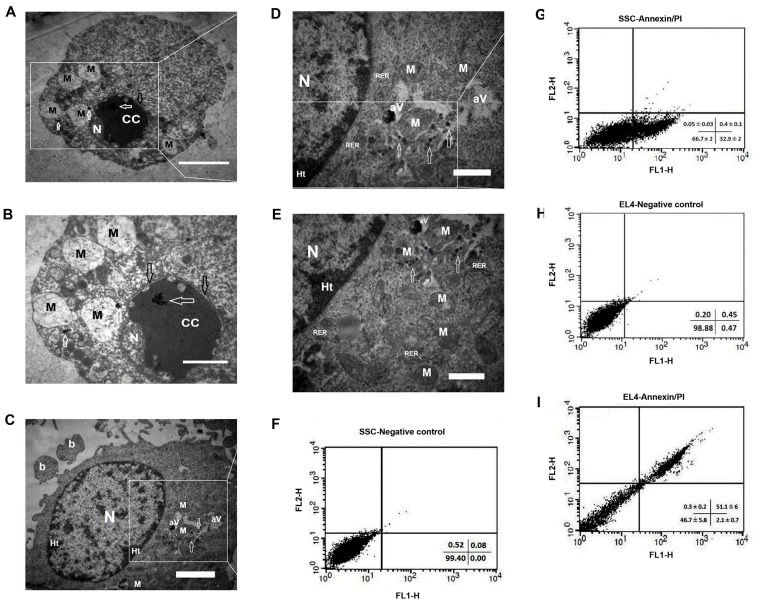
Ultrastructure and apoptosis in SSCs and EL4s after treatment with F-Si-GNR. Transmission electron micrographs from A, B. EL4s and C-E. SSCs after treatment with F-Si-GNR. Mitochondria were damaged and chromatin condensation was observed in EL4s also swelling of the nucleus membrane was observed in EL4s. The Rough endoplasmic reticulum (RER), autophagic vacuoles (aV) and very high mitochondria (M) were observed in SSCs. Swollen membrane (black arrows), F-Si-GNR (white arrows), nucleus (N), chromatin condensation (CC), Mitochondria (M), plasma membrane blebbing (b), (scale bar: A: 4 µm, B: 2 µm, C: 2 µm, D, E: 1 µm). Effects of F-Si-GNR administration on the apoptosis in F, G. SSCs, and H, I. EL4s determined by flow cytometry analysis. The diagram shows that after incubation with F-Si-GNR, necrotic SSCs and EL4 cells weren’t observed. F-Si-GNR; Folic acid-conjugated silica-coated gold nanorods.

The ultrastructural characteristics of EL4s and SSCs after treatment with F-Si-GNR were examined via TEM. The chromatin condensation was observed in the EL4s cells and the nucleus membrane was swollen. Swelling of the nuclear membrane is the first manifestation of injury to cells. Spherical mitochondria were also damaged (Fig .5A, B). The heterochromatin nucleus, plasma membrane blebbing and rough endoplasmic reticulum were obseved in SSCs. The mitochondria were found in relatively high numbers in the SSCs clusters whereas a few they were with damaged cristas. Gold nanorods were observed in the mitochondria and cytoplasm, and also the autophagic vacuoles were consist of nanoparticles (Fig .5C-E). 

### The apoptosis was measured using annexin V-FITC 

The apoptosis detection kit. The apoptotic rates of the EL4s (51.1 ± 6) were significantly higher than SSCs (Fig .5F, G) (32.9 ± 2, P<0.001). Also, after incubation with F-Si-GNR, necrotic SSCs and EL4s weren’t observed. Necrotic cells were positive for PI and negative for annexin V-FITC.

## Discussion

Since cancers, and especially testicular cancer, affects male fertility in many ways, an increase in the survival of male cancer patients of the fertile age has become a new challenge to male fertility. Cancer treatment, including radiation therapy, chemotherapy, and surgery, can be temporary and also have permanent harmful effects on male fertility (27). The isolation of cancer cells from healthy cells (germ cells) is a great challenge. Nowadays, the process of isolating testicular germ cells from malignant cells while avoiding contamination is in progress (26). So far, there are several techniques used to separate tumor cells from normal cells, including MACS and FACS-based sorting strategies and additional sorting techniques that avoid contamination of harmful cancer cells (9, 10). 

Nanotechnology has made a major stride in selective cancer targeting. They can be designed for targeting the favorable cells by changing various modifications of NPs such as their shape, size, physical and chemical properties (28). Gold NPs have a very high potential for cancer therapy based on their light absorption and scattering properties. NPs cause intracellular oxidative stress by disrupting the balance between antioxidative and oxidative processes. Research shows that some NPs can produce reactive oxygen species (ROS) which cause inflammation and even cell death (29). GNRs can be observed in many various shapes but most notably they are seen as nanorods and spherical clusters. Wang et al. (30) determined that nanorods are more cytotoxic than spherical gold nanomaterials to human HeCaT keratinocytes. GNRs support longitudinal plasmon resonances at NIR modulation with better quality factors than those of spherical gold NPs in the same resonance modulation (31), and they are extremely effectual at converting light energy into heat, especially if embedded in media of low thermal conductance (32). However, by themselves, the gold NPs desire to aggregate in solution and can smelt under laser irradiation. Silica coating is one of the golden functionalization tools that has been proven to increase the consistency of gold NPs, both thermodynamically and chemically (20, 21). The superior consistency with silica coating makes it the best choice for many applications.

Gold NPs can bind to antibodies and molecular ligands and they are suitable for medical applications (14, 18). According to Mehdizadeh et al. (19) studies, we used folate as a targeting ligand for gold NPs. Folate is transmitted in healthy cells and cancer cells by folate receptors on the cell membrane. Folate synthesizes thymine by dihydrofolate reductase in the cytoplasm of cells, so these cells regulate the presence of folate receptors on their surface. Because DNA synthesis and cell division are dependent on the presence of folate, a cancer cell needs a lot more folate than a healthy cell (22). Receptors of folate are located in caveolae on the cell membrane. After folate attachment to their receptors, it is internalized into the cytoplasm through the endocytic pathway (33). Previous studies have confirmed that folate-receptors are highly overexpressed on the surface of tumor cell types (17).

Li et al. (34) found that gold NPs functionalized with folate are selectively internalized into cells expressing folate receptor. Other studies showed that the increase of cytotoxicity for FR-targeted gold NPs loaded with doxorubicin in FR-expressing cells related to FR- mediated endocytosis (35). The benefits of synthesis of folate-functionalized gold NPs loaded with curmin (35) or cisplatin (36) as a chemotherapeutic cargo and resultant increase in cellular uptake of FR-targeted gold NPs has been reported. Zhang et al. (37) showed that superparamagnetic NPs conjugated with folate have better uptake in tumor cells. Mansoori et al. (38) investigated cell death in HeLa (high level of folate receptor expression) and MCF-7 (low level of folate receptor expression) cells. Their results also showed that uptake of folate-conjugated gold NPs in HeLa cells were more than for MCF-7 cells and that this difference was related to the number of folate receptors on the surface of the cell. Also, in another study, Xia et al. (22) used F-Si-GNRs on HeLa cells and A549 cells. The results indicated that more F-Si-GNRs were uptaken into HeLa cells via receptor-mediated endocytosis as compared to A549 cells. 

Here, in this study, we described a novel approach for elimination of cancerous cells from SSCs with treatment by F-Si-GNRs. We isolated SSCs from 3-6-day-old mice. To confirm the identity of these cells, RT-PCR using spermatogonial and germ cell markers was performed. SSCs expressed *Itgα6, Gfrα1, Itgß1, Oct4, Plzf* and *Mvh* markers and our findings are in line with results of previous research (25). SSCs demonstrated colonies by their morphology and they had a regular round nuclei that are similar to those found in other research (11, 25). EL4 cells were non-adherent and maintain their homogeneity. The margins of these cells were irregular. It should be noted that these cells don’t form colonies and have a high proliferation rate. Also, evaluation by flow cytometry showed that *Plzf* and *H-2kb* markers are expressed in SSCs and EL-4 cells, respectively.

In order to confirm tumorigenicity, EL4 cells were transplanted through the efferent ductus and into the seminiferous tubules of azoospermia mice. After transplantation, histological evaluations confirmed that EL4s can produce a tumor *in vivo*. Our findings are consistent with other studies (11).

In the present research, the survival of EL4s and SSCs after treatment with F-Si-GNRs was assessed using the MTT proliferation test. For dose response, we used multiple doses of F-Si-GNRs that consisted of 25, 50, 75, 100, 125 and 140 µM for 6 hours. Our study identified that cell death increased with an increase in the quantity of GNRs. In this study, we tried to find an effective dose of F-Si-GNRs for the elimination of EL4s, while maintaining SSC health and viability. The results of MTT assay showed that the optimal mean dose for the highest cell death in EL4s and lowest in SSCs is 100 µM of F-Si GNRs for a 6 hour incubation period.

Our results demonstrated that cytotoxicity of F-Si- GNRs increased in EL4 cells in comparison to SSCs. Similar to other studies, the increase in cytotoxicity is related to FR-mediated endocytosis and following uptake of F-Si-GNRs in tumor cells (22, 38). Moreover, the present study shows that different doses of F-Si-GNRs have concentration-dependent cytotoxic effects on EL4s and germ cells. The size of the NPs was found to play a crucial role in both the rate and extent of cellular uptake. Pan et al. (29) showed that toxicity of gold NPs are size- dependent. In our study, the size of the F-Si-GNRs was 20.43 ± 2.18 nm in length and 5.55 ± 1.56 nm in width. The thickness of the silica layer coating around the GNRs was 2.56 ± 0.62 nm, and this resulted in more toxicity compared to other studies (23, 24).

After incubation of SSCs and EL4s with F-Si-GNRs, apoptosis evaluation was performed using an annexin V-FITC apoptosis detection kit. The results showed that apoptotic rates of the EL4s were significantly higher than SSCs and this finding is similar to other research (11). This means that the numbers of folate receptors on the surface of EL4 cells are more abundant than for SSCs. After internalization, F-Si-GNRs were taken up by lysosomes. The lysosomal membrane is protected from acidic hydrolases by specific expression of lysosomal membrane proteins (39). The lysosomes were heavily disrupted and further damaged the mitochondrial membranes. Mitochondrial damage further activated the apoptosis-associated signaling pathways. In this research, electron microscopy studies showed F-Si-GNRs after cellular internalization and illustrated how these cause damage to the mitochondria, which is consistent with other studies (40).

## Conclusion

Here, we report the synthesis and characterization of folate conjugated silica modified GNRs and their *in vitro* effects on the viability of SSCs and EL4s. In addition, our results indicated that EL4s had a greater amount of uptake of F-Si-GNRs as compared to SSCs, and this was related to the amount of folate recepter that was present on the cells. The obtained results support the use of the optimal dose of F-Si-GNRs as a useful approach for treating testicular cancer. We anticipate that this NPs will have great potential for the development of therapies for clinical patients with cancer in near future. 
